# Which placebo to cure depression? A thought-provoking network meta-analysis

**DOI:** 10.1186/1741-7015-11-230

**Published:** 2013-10-25

**Authors:** Florian Naudet, Bruno Millet, Philippe Charlier, Jean Michel Reymann, Anne Solène Maria, Bruno Falissard

**Affiliations:** 1 INSERM U669, Maison de Solenn, 97 Boulevard de Port Royal, 75679 Paris Cedex 14, France; 2 Université de Rennes 1, EA-4712 Behavior and Basal Ganglia Unit, Rennes, France; 3 Centre Hospitalier Guillaume Régnier, Service Hospitalo-Universitaire de Psychiatrie, Rennes, France; 4 Department of Forensic Medicine and Pathology, University Hospital R. Poincaré (AP-HP, UVSQ), Garches, France; 5 Laboratory of Medical Ethics, University of Paris 5, 45 St Pères Street, 75006 Paris, France; 6 Centre d’Investigation Clinique CIC-P INSERM 0203, Hôpital de Pontchaillou, Centre Hospitalier Universitaire de Rennes et Université de Rennes 1, Rennes, France; 7 Laboratoire de Pharmacologie Expérimentale et Clinique, Faculté de Médecine, CS34317, 2 Avenue du Pr Léon Bernard, 35043 Rennes, France; 8 Université Paris-Sud and Université Paris Descartes, UMR-S0669, Paris, France; 9 AP-HP, Hôpital Paul Brousse, Département de santé publique, Villejuif, France

**Keywords:** Antidepressants, Placebo, Major depressive disorder, Meta-analysis, Publication bias

## Abstract

**Background:**

Antidepressants are often considered to be mere placebos despite the fact that meta-analyses are able to rank them. It follows that it should also be possible to rank different placebos, which are all made of sucrose. To explore this issue, which is rather more epistemological than clinical, we designed an unusual meta-analysis to investigate whether the effects of placebo in one situation are different from the effects of placebo in another situation.

**Methods:**

Published and unpublished studies were searched for by three reviewers on Medline, the Cochrane Library, Embase, clinicaltrials.gov, Current Controlled Trial, in bibliographies, and by mailing key organizations. The following studies in first-line treatment for major depressive disorder were considered to construct an “evidence network”: 1) randomized controlled trials (RCTs) versus placebo on fluoxetine, venlafaxine and 2) fluoxetine versus venlafaxine head-to-head RCTs.

Two network meta-analyses were run to indirectly compare response and remission rates among three different placebos: 1) fluoxetine placebo, 2) venlafaxine placebo, and 3) venlafaxine/fluoxetine placebo (that is, placebo compared to both venlafaxine and fluoxetine). Publication biases were assessed using funnel plots and statistically tested.

**Results:**

The three placebos were not significantly different in terms of response or remission. The antidepressant agents were significantly more efficacious than the placebos, and venlafaxine was more efficacious than fluoxetine. The funnel plots, however, showed a major publication bias.

**Conclusion:**

The presence of significant levels of publication bias indicates that we cannot even be certain of the conclusion that sucrose equals sucrose in trials of major depressive disorder. This result should remind clinicians to step back to take a more objective view when interpreting a scientific result. It is of crucial importance for their practice, far more so than ranking antidepressant efficacy.

## Background

The history of medicine is closely linked to the history of placebos. Pre-scientific medicine was based on many bizarre and ineffective medical interventions and on the belief that such treatments were effective [1]. Placebo was used for the first time as a control in 1784 to debunk the healing claims of mesmerism [2], and it became a standard control in experimental procedures in the second half of the 20^th^ century. Randomized controlled trials (RCTs) against placebo have enabled major progress in modern medicine. Nevertheless, these studies have limitations in terms of external validity and even internal validity, and antidepressant literature on major depressive disorder is a striking example of these limitations: some practitioners and researchers [3] consider that most of the antidepressant efficacy reflects simply the placebo effect, especially for depression in patients with mild or moderate symptoms [4,5]. However, many patients are satisfied with these treatments, many clinicians trust them and use them, and a large part of discussions during medical staff meetings is devoted to the choice of the right sort of antidepressant drug [6]. Recently, Cipriani *et al.* in a multiple-treatment meta-analysis, ranked 12 new-generation antidepressants [7] to address this question.

This state-of-the-art raises a fundamental question: if much of the effect of antidepressants is attributable to the placebo effect and if it is possible to rank antidepressants, then it should also be possible to rank different placebos, which are all made of sucrose. In a more global perspective, it questions whether or not we can be certain about anything in psychiatry (or, indeed, in medicine), and, in particular, whether the evidence that we usually rely on provides us with a reasonable degree of certainty about the nature and effectiveness of our practices. We set about investigating this question, which is rather more epistemological than clinical, by investigating whether the effects of placebo in one situation are different from the effects of placebo in another situation. We thus designed an unusual meta-analysis on aggregated data which allows us to examine the apparently incontrovertible fact that sucrose equals sucrose by comparing the placebos of two famous antidepressant blockbusters: 1) fluoxetine, one of the first selective serotonin reuptake inhibitors available on the market, which has become a reference drug, and 2) venlafaxine, a serotonin-norepinephrine reuptake inhibitor.

## Methods

### Eligibility criteria

#### Types of participants

We reviewed studies involving adults (age 18 and over) with a diagnosis of major depressive episode (DSM IV, DSM IV-R, DSM III, DSM III-R, ICD 10, Feighner criteria, Research Diagnostic Criteria). Studies involving patients with other psychiatric or medical comorbidity were considered only when it was not an explicit inclusion criterion for the study. Studies involving patients with a diagnosis of anxious depression were also considered.

Studies involving more than 20% subjects with bipolar disorder were excluded, as were studies exclusively involving patients with seasonal affective disorder, post-partum depression, postmenopausal depression, atypical depression, dysthymia and studies involving elderly patients.

#### Types of intervention(s)

Our primary aim was to compare placebo arms. We focused our attention on three different placebos: 1) fluoxetine placebo (FLUp, where placebo was compared to fluoxetine), 2) venlafaxine placebo (VENLAFp, where placebo was compared to venlafaxine), and 3) venlafaxine/fluoxetine placebo (FLU/VENLAFp, where placebo was compared to both venlafaxine and fluoxetine), which were obviously compared with the corresponding antidepressants in oral mono-therapy in major depressive disorder first line acute treatment.

#### Types of outcomes

Response was chosen as the primary outcome. Remission was chosen as a secondary outcome. These outcomes are the most consistently reported estimates of acute-treatment efficacy. Response was defined as the proportion of patients who had a reduction of at least 50% from the baseline score on the Hamilton Depression Rating Scale (HDRS) [8] or the Montgomery-Åsberg Depression Rating Scale (MADRS) [9]. Remission was defined as the proportion of patients who had a HDRS score ≤7 or a MADRS score ≤12.

When trials reported results from both rating scales, we extracted data from the scale considered in the study as the primary outcome.

#### Types of studies

In this review were included 1) randomized controlled trials of fluoxetine or venlafaxine against placebo and 2) head-to-head trials of fluoxetine versus venlafaxine with or without placebo control. All studies were conducted from January 1989 to July 2009. Only study reports in English, French and Spanish language were considered.

### Search strategy

We used the search strategy from an earlier paper [10] on venlafaxine and fluoxetine to conduct this meta-analysis on aggregated data.

Eligible studies were identified from PubMed/Medline, the Cochrane library and Embase, including congress abstracts. A three-step search was used for each component of this review. In a first step, an initial search on Medline was carried out in order to refresh optimal search terms and include possible changes in the databases. The search terms used were double-checked before starting the main search. In a second step, all keywords identified were used to search all the above-mentioned databases. A third search was undertaken by searching the reference lists of articles identified. Initial keywords used were: “Depressive Disorder NOT Depression, Postpartum NOT Seasonal Affective Disorder”; “Antidepressive Agents”; “Fluoxetine”; “Venlafaxine”. In addition, manual searches of articles were performed in previous meta-analyses.

Unpublished studies were searched for by communication with key organizations such as the Food and Drug Administration (FDA) and the European Medicines Agency (EMEA), and key researchers in the area. A search on clinicaltrials.gov and Current Controlled Trial was performed.

Authors of abstracts or meta-analysis were contacted for further information and were asked for references of the studies when needed. If no response was obtained to a first solicitation, they were then contacted a second time.

### Study selection

Eligibility assessment was performed independently in a blinded standardized manner by two reviewers (NF and MAS). Disagreements were resolved by consensus or in consultation with a third reviewer (FB).

A comparison across the studies, checking author names, treatment comparisons, sample sizes and outcomes was performed to avoid duplicates and compilations of data from several reports of the same study.

### Assessment of methodological quality

Each paper was then assessed for methodological quality prior to inclusion in the review using an appropriate standardized critical appraisal instrument from the Joanna Briggs Institute [11].

### Data collection

A data extraction sheet based on the Cochrane Handbook for Systematic Reviews of Interventions’ guidelines (Version 5.0.2, updated September 2009) [12] was used. One review author (NF) extracted these data from the studies included.

### Data analysis

The dichotomous outcomes used here are considered as robust outcome measures of treatment efficacy [7]. When these outcomes were not reported, the studies were excluded, and no imputation method was used. Responder and remitter data were extracted as the original study investigators analyzed the data, mainly using the LOCF (Last Observation Carried Forward) method.

#### Head-to-head direct evidence

We used visual inspection of the forest plots and the Q statistic [13] to investigate the possibility of statistical heterogeneity. In the absence of heterogeneity we performed pair-wise meta-analyses by synthesizing studies that compared the same interventions with a fixed-effects model (Mantel-Haenszel); in case of possible heterogeneity, we performed pair-wise meta-analysis with a random effect model (DerSimonian and Laird).

#### Network meta-analyses to enable indirect comparisons

In a second step, to compare indirectly the different placebos, and following recommendations by Glenny *et al.*[14], two network meta-analyses [15,16] were run. The dependent variables were 1) response and 2) remission; the treatment was considered as the explanatory variables (fluoxetine, venlafaxine, FLUp, VENLAFp and FLU/VENLAFp). Both fixed effect and random effect approaches were performed for each network meta-analysis; final models were selected by comparing a model fit criterion (Akaïke’s Information Criterion (AIC)). Results of these meta-analyses are the odds ratio (OR) between treatments with their 95% confidence interval and the statistical significance level of the comparison.

#### Risk of bias across studies

Publication bias was investigated graphically using funnel plots for each fixed effect meta-analysis. Funnel plot asymmetry was tested using the rank correlation test when there were at least 10 studies [17].

Analyses were performed using R [18] with libraries meta [19], rmeta [20] and lme4 [21] (lmer function, family = binomial). Results are presented according to PRISMA (Preferred Reporting Items for Systematic Reviews and Meta-Analyses) statements [22].

## Results

### Study selection

**Figure 1:**
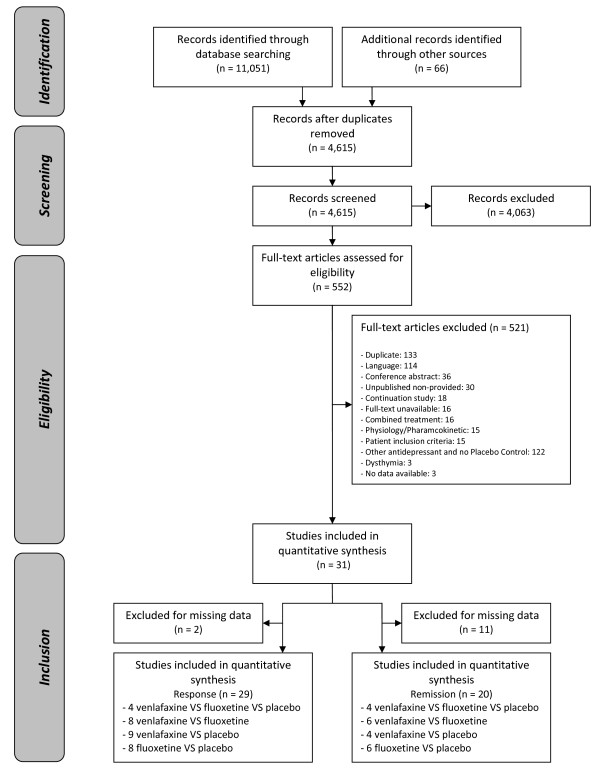
Flow diagram.

The search of Medline, Cochrane and Embase databases provided a total of 11,051 citations. An additional 66 studies were identified by manual search. After adjusting for duplicates, 4,615 remained. Of these, 4,063 studies were discarded because, after review of the abstracts, it appeared that these papers did not meet the criteria. A total of 114 studies were excluded because of the language (among which 97 were in Chinese). Of 33 unpublished relevant studies identified, only 3 were made available by pharmaceutical firms. Thirty-one studies were included in the quantitative review. A flow-chart detailing the study selection process is given in Figure 1.

### Study characteristics and risk of bias within studies

A summary of the methodologies of these 31 studies is given in Table 1. The quality of the different studies is presented as a table in a web appendix (Additional file 1: Table S1). The “response” analysis involved 29 RCTs including 7,459 participants and the “remission” analysis involved 20 RCTs including 5,096 participants.

**Table 1:** Summary of study methodology

	**V vs F vs *P *** **(n = 4)**	**V vs F (n = 8)**	**V vs *P *** **(n = 10)**	**F vs P (n = 9)**	**Total (n = 31)**
**Year**	(1999, 2003, 2004, 2009)	(1994, 1997, 1998, 2000, 2007)	(1993, 1996, 1997, 1998, 2004)	(1991, 1998, 2002, 2004, 2005)	(1991, 1997, 1999, 2003, 2009)
**Anxious depression (No)**	3 (75%)	7 (88%)	10 (100%)	8 (89%)	28 (90%)
**Study duration (weeks)**	(6, 6, 8, 12)	(6, 8, 9, 12, 12)	(4, 6, 8, 11, 13)	(4, 6, 8, 12, 13)	(4, 6, 8, 12, 13)
**Number of follow-up visits**	(6, 7, 8, 8)	(6, 6, 7, 8, 9) (NA = 1)	(5, 6, 7, 8, 8)	(3, 8, 9, 9, 9) (NA = 1)	(3, 6, 7, 8, 9) (NA = 2)
**Industry sponsorship (yes)**	4 (100%)	8 (100%)	10 (100%)	8 (100%) (NA = 1)	30 (100%) (NA = 1)
**Exclusion of placebo responders (yes)**	4 (100%)	5 (63%)	10 (100%)	6 (75%) (NA = 1)	25 (83%) (NA = 1)
**Patient type**			(NA = 1)	(NA = 1)	(NA = 2)
Inpatients	1 (25%)	2 (25%)	1 (11%)	1 (12.5%)	5 (17%)
Outpatients	3 (75%)	5 (63%)	7 (78%)	6 (75%)	21 (73%)
Outpatients in primary care	0 (0%)	1 (12%)	1 (11%)	1 (12.5%)	3 (10%)
**Scale used**					
HDRS	4 (100%)	5 (63%)	4 (40%)	8 (89%)	23 (74%)
MADRS	0 (0%)	3 (37%)	6 (60%)	1 (11%)	8 (26%)
**Type of analysis**					
ITT with LOCF	4 (100%)	8 (100%)	10 (100%)	8 (89%)	30 (97%)
Mixed model	0 (0%)	0 (0%)	0 (0%)	1 (11%)	1 (3%)
**Initial severity (HDRS score **≥**25)**	3 (75%)	5 (63%)	6 (60%)	3 (37.5%) (NA = 1)	17 (57%) (NA = 1)

n, number of studies. Results are presented in (minimum, first quartile, median, third quartile, maximum) for quantitative data except for V vs F vs P where all the data are given. Qualitative data are presented as a number (percentage). NA represents the number of studies where data were unavailable.

V vs F vs P: studies comparing venlafaxine to fluoxetine to placebo.

V vs F: studies comparing venlafaxine to fluoxetine.

V vs P: studies comparing venlafaxine to placebo.

F vs P: studies comparing fluoxetine to placebo.

HDRS, Hamilton Depression Rating Scale; MADRS, Montgomery-Åsberg Depression Rating Scale.

### Results from individual studies and synthesis of results

#### Head-to-head direct evidence

Using the Q statistic, no significant heterogeneity was detected for fluoxetine versus placebo, venlafaxine versus placebo or for fluoxetine versus venlafaxine in the response and remission meta-analyses. Nevertheless, as visual inspection of the forest plot suggested that heterogeneity could not be totally excluded, and as heterogeneity tests are often under-powered [23], we ran pair-wise fixed effects and random effects models which found the same results.

**Figure 2:**
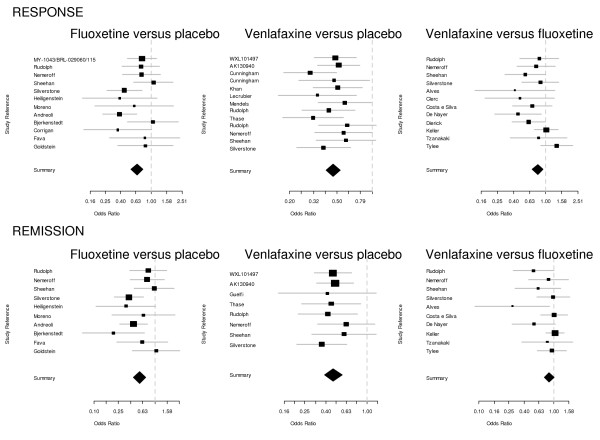
**Forest plots.** Forest plots present head-to-head meta-analyses using fixed-effects model for response and remission comparing 1) placebo vs fluoxetine, 2) placebo vs venlafaxine and 3) fluoxetine vs venlafaxine.

Results of the head-to-head direct meta-analysis using a fixed-effects model are presented in Figure 2 and in the web-appendix (Additional file 1: Table S2) and results of random-effects model are presented in the web appendix (Additional file 1: Table S2 and Additional file 1: Figure S1): regarding treatment response, placebo appeared less effective than both fluoxetine and venlafaxine. Fluoxetine appeared less effective than venlafaxine.

#### Network meta-analyses to enable indirect comparisons

**Figure 3:**
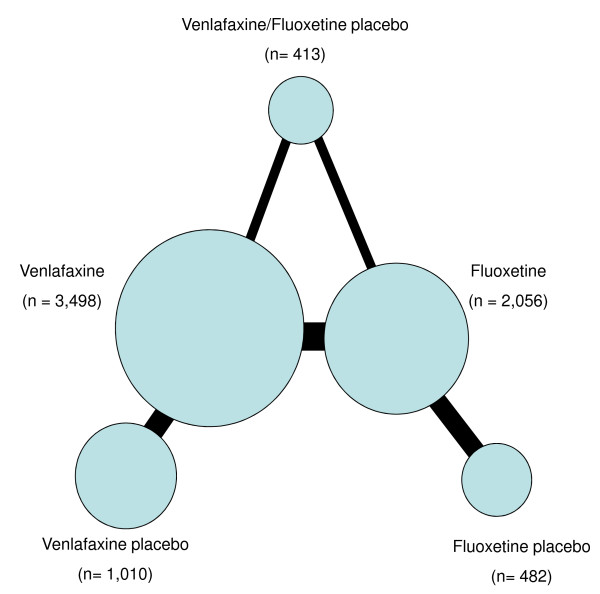
**Summary of the evidence used in the network meta-analysis for response rate.** The thickness of the lines is proportional to the number of trials comparing each pair of treatments, and the size of each node is proportional to the number of randomized participants (n = sample size). The network of eligible comparisons for remission analysis was similar (with fewer patients and comparisons).

Figure 3 shows all the available evidence for the meta-analysis concerning the response. Concerning the two network meta-analyses (response and remission), AIC (presented in the e-appendix, Additional file 1: Table S3) was in favor of the selection of the fixed effect network meta-analysis (which fits the data better).

Table 2 summarizes the results of these two fixed-effect network meta-analyses concerning response and remission. The three placebos were not significantly different in terms of response (*P* = 0.33 for FLU/VENLAFp versus VENLAFp, *P* = 0.50 for FLU/VENLAFp versus FLUp and *P* = 0.85 for VENLAFp versus FLUp) or remission (*P* = 0.88 for FLU/VENLAFp versus VENLAFp, *P* = 0.66 for FLU/VENLAFp versus FLUp and *P* = 0.76 for VENLAFp versus FLUp). Antidepressant agents were significantly more efficacious than placebos and venlafaxine was more efficacious than fluoxetine. Results of the random effect meta-analysis were coherent and are presented in the web-appendix (Additional file 1: Table S4).

**Table 2:** Results of the network meta-analyses

**FLUp**		**1.03**	**[0.75 to 1.41]**	**0.88**	**[0.63 to 1.25]**	**0.58**	**[0.46 to 0.74]**	**0.46**	**[0.35 to 0.60]**
0.94	[0.61 to 1.43]	**VENLAFp**		**0.86**	**[0.64 to 1.16]**	**0.57**	**[0.46 to 0.70]**	**0.45**	**[0.37 to 0.52]**
0.91	[0.59 to 1.39]	0.97	[0.66 to 1.42]	**FLU/VENLAFp**		**0.66**	**[0.51 to 0.84]**	**0.51**	**[0.40 to 0.66]**
0.51	[0.37 to 0.71]	0.55	[0.41 to 0.73]	0.57	[0.42 to 0.76]	**FLU**		**0.79**	**[0.68 to 0.91]**
0.44	[0.31 to 0.63]	0.47	[0.37 to 0.61]	0.49	[0.36 to 0.65]	0.86	[0.74 to 1.00]	**VENLAF**	

Results of the network meta-analyses are the Odds ratio (OR) between treatment in the column and treatment in the row with their 95% confidence interval. For response (in bold), OR higher than one favor the treatment indicated in the row. For remission (not in bold), OR higher than one favor the treatment indicated in the column. To obtain OR for comparisons in the opposite direction, reciprocals should be taken. Significant results are underscored.

### Risk of bias across studies

**Figure 4:**
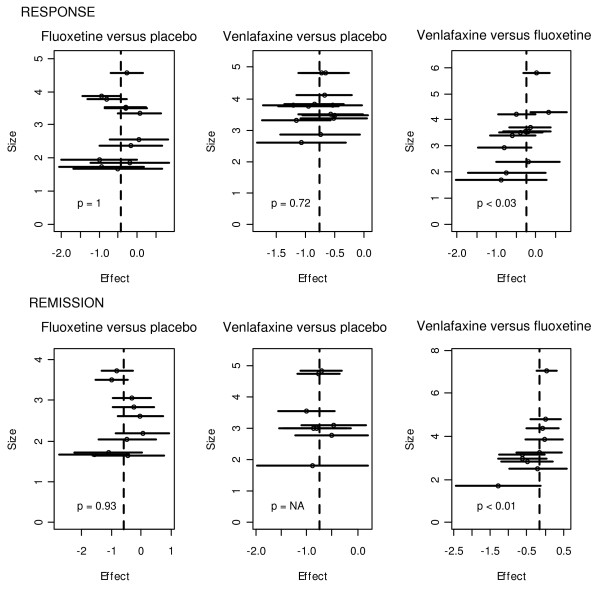
**Funnel plots.** Six funnel plots were drawn: three for response and three for remission, for each head to head direct meta-analysis. The *P*-value given for each funnel is the result of the rank correlation test of funnel plot asymmetry. NA, test not applicable (<10 studies in the meta-analysis).

Six funnel plots were drawn (three for response and three for remission for each head-to-head direct meta-analysis, Figure 4). The funnel plots of the meta-analysis comparing venlafaxine to fluoxetine showed some asymmetry (*P* <0.03 for response and *P* <0.01 for remission). The asymmetry was less striking for funnel plots of meta-analyses for both active treatments versus placebo.

## Discussion

### Summary of evidence

The three placebos considered were not significantly different in terms of response or remission. Antidepressant agents were significantly more efficacious than placebos, and venlafaxine was more efficacious than fluoxetine. This was coherent with previous meta-analyses [7,24]. Thus, venlafaxine placebo appears as a “me-too” placebo without greater benefit in relation to fluoxetine placebo and/or fluoxetine and venlafaxine placebo.

Since, obviously, no direct evidence of well-powered, randomized, direct-comparisons exist, indirect comparisons were the only option for comparing the three different placebos. Such indirect evidence is not totally the same as direct evidence and in some cases it has been shown that indirect comparisons led to conflicting results as compared with direct evidence. Such a paradox has been recently shown concerning antidepressants in a recent paper comparing citalopram with its “me-too” escitalopram which found an inconsistency between direct evidence (showing a superiority of escitalopram) and indirect evidence (which did not find any significant difference) [25]. Additionally, Song *et al.* have suggested that in some cases indirect evidence is less biased than direct evidence [26]. Moreover, the validity of indirect comparison is dependent on the distribution of relative treatment effect modifiers across different comparisons [27]. In our study, initial severity (HDRS score ≥25) could be an important effect modifier [4,5] but despite slight variations, its distribution seemed well-balanced across the different direct comparisons (that is, there was no systematic difference in its distribution between the different direct comparisons).

Nevertheless, results of our indirect comparisons were consistent with the implicit conception that if the three placebos (all made of the same sucrose) were compared in a double blind randomized trial, no difference would be observed. These results are good news indeed: 1) for the supporters of placebo because they conclude that the choice of the placebo is not really important, 2) for the supporters of antidepressants because antidepressants prove superior to placebos, and 3) for supporters of rationality because a validated method has not led to invalid conclusions, that is, we have managed to conclude that “sucrose equals sucrose” in the treatment of major depressive disorder, which is wholly reassuring. Yet significant limitations question these findings.

### Limitations

The quality of a meta-analysis is linked to the quality of the individual studies included [28]. In this respect, the National Institute for Health and Clinical Excellence (NICE) guideline on the treatment and management of depression in adults [29] advises caution in the application of results from RCTs and meta-analyses in routine practice. In particular, our model is based on the postulate that all placebo responders should be antidepressant responders (additive model). This key assumption has never been proved to be true. Indeed, antidepressant response and placebo response could be independent or at least substantially overlapping phenomena (non-additive model), with four different types of patients: 1) placebo-only responders, 2) treatment-only responders, 3) placebo and treatment responders, and 4) never responders [30]. Moreover, the classic logic of the randomized controlled trial casts the placebo effect as a negative foil for measuring therapeutic efficacy and a large amount of important information concerning placebo is not reported in these studies, such as the appearance of the medication (size, shape and color of the pills) [31].

As well, the indirect comparison may have had low statistical power. Even if no trend toward statistical significance was observed within the indirect comparisons of placebos, insignificant *P*-values never tell much about equivalence [32].

Above all, a publication bias and a selective outcome reporting bias might account for some of the effects we observed. The funnel plot for comparison between venlafaxine and fluoxetine shows some asymmetry in favor of venlafaxine. As “true heterogeneity” could not explain this result, and since studies on antidepressants generate substantial conflicts of interest (these drugs generate vast sales revenues), the result is very much open to suspicion of reporting bias [17]. Concerning comparisons between active antidepressants and placebo, no evidence was found for a publication bias, but as statistical tests for asymmetry typically have low power [17], this bias cannot be excluded. The bias is well known [33] and, for example, it has led to considering reboxetine as a serious antidepressant agent, whereas it is probably ineffective and potentially harmful [34]. It has been recently demonstrated that the selective reporting of studies in network meta-analysis of antidepressants biases estimates of relative treatment efficacy [35].

Various barriers were encountered in our meta-analytic quest for exhaustiveness: many trials were carried out in China and published in Chinese journals. Nevertheless, the quality of many of these studies could be expected to be poor [36], and excluding these trials, although it means a loss of randomized evidence, thus avoids other major biases.

Moreover - and this is probably the main problem - antidepressant research is completely controlled by the pharmaceutical industry [37]: 1) the firms that promoted some of the trials we identified refused to communicate results from these studies; 2) in this meta-analysis all the studies were sponsored by the pharmaceutical industry. Such studies have been shown to be more likely to demonstrate positive effects for the sponsor’s drug than independent studies [38].

### Perspectives

Thus, in view of these limitations, a reasonable measure of skepticism should discourage hasty conclusions. They illustrate the fact that every scientific result is uncertain and that it is difficult to be sure of an individual study conclusion, even if it explored something as patently obvious as “sucrose = sucrose”, however rigorous the method. Nevertheless, although published research findings can be erroneous [39], Science often generates representations that leave no room for skepticism. This is probably the most insidious pitfall in Evidence-Based Medicine; it does not concern the findings of Science, it concerns academics’ understanding of Science (the knowledge-producing activity). The present-day context of medicalization of modern society [40] implicitly dictates that scientific results should have the status of Truth. Concerning major depressive disorder, although it is likely to be untrue, clinicians and a great number of patients [41] strongly believe that antidepressant drugs target a specific biological state that produces depression [42] and the pleasing serotonin hypothesis is often taken as gospel [43].

As for the study by Cipriani *et al*., it was disputed with similar arguments to those set out in the present study [37,44-47] and its results were not replicated by Gartlehner *et al.*[48]. Moreover, whereas in our study insignificant *P*-values do not tell much about equivalence, in over-powered studies, like Cipriani’s study, statistically significant differences never tell much about clinically significant differences [32]. It is nevertheless mentioned in the NICE guideline [29], with some kind of double bind: qualitatively no recommendations for ranking antidepressants are made, but quantitatively special emphasis is placed on the study (Cipriani’s name is cited 23 times versus 3 times for Gartlehner, tables present the results, and so on). What is more, in day-to-day practice, clinicians generally consider that Cipriani’s study is solid evidence for choosing antidepressants when treating a patient with newly diagnosed depression [49]. Uncertainty is sometimes acknowledged theoretically, but not in clinical practice.

This epistemological position translates into the well-known anthropological observation that the hopes and expectations of the physician are just as crucial as those of the patient in the healing process [50]. This suggests that while the opposite may not be true, the best placebos to treat Major Depressive disorder could be antidepressants because they are believed to be effective, which is probably an important determinant in the placebo effect [51].

Nonetheless, we cannot simply assume that, because patients appear to improve on placebos in the short term, as we observe in randomized controlled trials, placebos have demonstrated the required cost-benefit balance. When one uses a treatment that relies on expectation, one must also be careful as to its possible harmful consequences, which could be linked with the corollary, disappointment. But here again this is an unsolved central question in Evidence Based Medicine.

## Conclusion

### Implications for research

As in pre-scientific medicine, modern physicians need to believe in the effectiveness of their techniques, and current medical literature, with its strengths and also its limitations, appears as the sophisticated way to generate such beliefs. It also raises the ethical issue of the dissemination of scientific evidence, for example, when editors permit reprints for the pharmaceutical industry intended to present results to doctors via a more commercial than epistemological approach, as was the case for the Cipriani study [52].

### Clinical implications

We did not find any superiority of one placebo over the other. However, a critical approach to our results prevents any firm conclusion on this apparently obvious result. This result should remind clinicians to step back to take a more objective view when interpreting a scientific result, keeping in mind that Science can never be actually sure that “sucrose = sucrose” in the treatment of major depressive disorder. It is of crucial importance for their practice, far more so than ranking antidepressant efficacy.

## Abbreviations

AIC: Akaïke’s information criterion; DSM III: Diagnostic and statistical manual of mental disorders, 3^rd^ edition; DSM III-R: Diagnostic and statistical manual of mental disorders, 3^rd^ edition, text revised; DSM IV: Diagnostic and statistical manual of mental disorders, 4^th^ edition; DSM IV-R: Diagnostic and statistical manual of mental disorders, 4^th^ edition, text revised; EMEA: European medicines agency; FDA: Food and drug administration; FLU/VENLAFp: Venlafaxine/fluoxetine placebo; FLUp: Fluoxetine placebo; HDRS: Hamilton depression rating scale; ICD 10: International classification of diseases, 10th Revision; LOCF: Last observation carried forward; MADRS: Montgomery-Åsberg depression rating scale; NICE: National institute for health and clinical excellence; OR: Odds ratio; PRISMA: Preferred reporting items for systematic reviews and meta-analyses; RCTs: Randomized controlled trials; VENLAFp: Venlafaxine placebo.

## Competing interests

There are no conflicts of interest regarding this paper. All authors have completed the Unified Competing Interest form at http://www.icmje.org/coi_disclosure.pdf (available on request from the corresponding author) and declare that no authors have support from any company for the submitted work. NF has relationships (board membership or Travel/accommodations expenses covered/reimbursed) with Servier, BMS, Lundbeck and Janssen who might have an interest in the work submitted in the previous three years. MB has relationships (consultancy and travel/accommodations expenses covered/reimbursed) with Janssen, BMS, Otsuka, Lundbeck, Lilly, Servier, Astra Zeneca, Medtronics, Syneïka and has received grants for research from Medtronic, Lilly and Astra Zeneca in the previous three years. CP, RJM and MAS have no relationships with any company that might have an interest in the submitted work in the previous three years. FB has relationships (board membership or consultancy or payment for manuscript preparation or Travel/accommodations expenses covered/reimbursed) with Sanofi-Aventis, Servier, Pierre-Fabre, MSD, Lilly, Janssen-Cilag, Otsuka, Lundbeck, Genzime, Roche and BMS who might have an interest in the work submitted in the previous three years. NF, CP, MAS, RJM, FB and their spouses, partners or children have no financial relationships that may be relevant to the submitted work; MB’s spouse is an employee of Janssen. None of the authors has any non-financial interests that may be relevant to the submitted work.

## Authors’ contributions

FN conceived and designed the experiments, performed the experiments, analyzed the data and wrote the paper. FN had full access to all of the data in the study and takes responsibility for the integrity of the data and the accuracy of the data analysis. BM, PC and JMR revised the paper critically for important intellectual content. ASM performed the experiments. BF participated in the design of the experiments, performed the experiments and revised the paper critically for important intellectual content. All authors read and approved the final manuscript.

## Pre-publication history

The pre-publication history for this paper can be accessed here:

http://www.biomedcentral.com/1741-7015/11/230/prepub

## Supplementary Material

Additional file 1: Table S1 Studies included and their quality assessment according to the standardized critical appraisal instrument from the Joanna Briggs Institute. **Table S2.** Head to head meta-analyses (using fixed-effects model (Mantel-Haenszel = MH) and random-effects (DerSimonian and Laird = DSL)) of response and remission between 1) placebo vs fluoxetine, 2) placebo vs venlafaxine and 3) fluoxetine vs venlafaxine. **Table S3.** Akaïke’s Information Criterion (AIC) for fixed effect and random effects meta-analyses of response and remission. **Table S4.** Odds ratio (OR) of response and remission between fluoxetine placebo, venlafaxine placebo, fluoxetine/venlafaxine placebo, fluoxetine and venlafaxine. **Figure S1.** Forest plot presenting head-to-head meta-analyses using random-effects model for response and remission comparing 1) placebo vs fluoxetine, 2) placebo vs venlafaxine and 3) fluoxetine vs venlafaxine. Click here for file
