# Supplementary material for: Which placebo to cure depression? A thought-provoking network meta-analysis
Source: BMC Med. 2013 Oct 25;11:230. doi: 10.1186/1741-7015-11-230 (PMC3819667; doi:10.1186/1741-7015-11-230)
Supplement: Additional file 1: Table S1 — Studies included and their quality assessment according to the standardized critical appraisal instrument from the Joanna Briggs Institute. Table S2. Head to head meta-analyses (using fixed-effects model (Mantel-Haenszel = MH) and random-effects (DerSimonian and Laird = DSL)) of response and remission between 1) placebo vs fluoxetine, 2) placebo vs venlafaxine and 3) fluoxetine vs venlafaxine. Table S3. Akaïke’s Information Criterion (AIC) for fixed effect and random effects meta-analyses of response and remission. Table S4. Odds ratio (OR) of response and remission between fluoxetine placebo, venlafaxine placebo, fluoxetine/venlafaxine placebo, fluoxetine and venlafaxine. Figure S1. Forest plot presenting head-to-head meta-analyses using random-effects model for response and remission comparing 1) placebo vs fluoxetine, 2) placebo vs venlafaxine and 3) fluoxetine vs venlafaxine. [file 1741-7015-11-230-S1.doc]

**Online-only material**

**e-table 1**

**e-table 2**

**e-table 3**

**e-table 4**

**e- figure 1**

| **Author** | **Year** | **Was the assignment to treatment groups truly random?** | **Were participants blinded to treatment allocation?** | **Was allocation to treatment groups concealed from the allocator?** | **Were the outcomes of people who withdrew described and included in the analysis?** | **Were those assessing outcomes blind to the treatment allocation?** | **Were the control and treatment groups comparable at entry?** | **Were groups treated identically other than for the named interventions?** | **Were outcomes measured in the same way for all groups?** | **Were outcomes measured in a reliable way?** | **Was appropriate statistical analysis used?** |
| --- | --- | --- | --- | --- | --- | --- | --- | --- | --- | --- | --- |
| **AK130940** | 2005 | 😐 | ☺ | ☺ | ☹ | ☺ | 😐 | 😐 | ☺ | ☺ | ☺ |
| **Alves** | 1999 | ☺ | ☺ | ☺ | 😐 | ☺ | ☺ | 😐 | ☺ | ☺ | ☺ |
| **MY-1043/BRL-029060/115** | 1991 | 😐 | ☺ | ☺ | ☹ | ☺ | 😐 | 😐 | ☺ | ☺ | ☹ |
| **Andreoli** | 2002 | 😐 | ☺ | ☺ | ☹ | ☺ | ☺ | ☺ | ☺ | ☺ | ☺ |
| **Bjerkenstedt** | 2004 | 😐 | ☺ | ☺ | ☹ | ☺ | ☺ | 😐 | ☺ | ☺ | ☹ |
| **Clerc** | 1994 | 😐 | ☺ | ☺ | 😐 | ☺ | ☺ | 😐 | ☺ | ☺ | ☹ |
| **Corrigan** | 2000 | 😐 | ☺ | ☺ | ☹ | ☺ | 😐 | 😐 | ☺ | ☺ | ☹ |
| **Costa E Silva** | 1998 | 😐 | ☺ | ☺ | 😐 | ☺ | ☺ | 😐 | ☺ | ☺ | ☹ |
| **Cunningham** | 1997 | 😐 | ☺ | ☺ | ☹ | ☺ | ☺ | 😐 | ☺ | ☺ | ☹ |
| **Cunningham** | 1994 | 😐 | ☺ | ☺ | ☹ | ☺ | ☺ | 😐 | ☺ | ☺ | ☹ |
| **Dierick** | 1996 | 😐 | ☺ | ☺ | ☹ | ☺ | ☺ | ☺ | ☺ | ☺ | ☹ |
| **Fava** | 2005 | 😐 | ☺ | ☺ | ☹ | ☺ | 😐 | 😐 | ☺ | ☺ | ☹ |
| **Goldstein** | 2002 | 😐 | ☺ | ☺ | ☺ | ☺ | ☺ | 😐 | ☺ | ☺ | ☺ |
| **Guelfi** | 1995 | 😐 | ☺ | ☺ | ☹ | ☺ | ☺ | ☺ | ☺ | ☺ | ☹ |
| **Heiligenstein** | 1993 | 😐 | ☺ | ☺ | ☹ | ☺ | ☹ | 😐 | ☺ | ☺ | ☹ |
| **Khan** | 1998 | 😐 | ☺ | ☺ | ☹ | ☺ | ☺ | 😐 | ☺ | ☺ | ☹ |
| **Lecrubier** | 1997 | 😐 | ☺ | ☺ | ☹ | ☺ | ☺ | 😐 | ☺ | ☺ | ☹ |
| **Mendels** | 1993 | 😐 | ☺ | ☺ | ☹ | ☺ | ☺ | 😐 | ☺ | ☺ | ☹ |
| **Moreno** | 2005 | 😐 | ☺ | ☺ | ☹ | ☺ | ☹ | 😐 | ☺ | ☺ | ☺ |
| **Nemeroff** | 2007 | 😐 | ☺ | ☺ | ☹ | ☺ | ☺ | 😐 | ☺ | ☺ | ☹ |
| **Rudolph** | 1998 | ☺ | ☺ | ☺ | ☹ | ☺ | ☺ | 😐 | ☺ | ☺ | ☹ |
| **Rudolph** | 1999 | ☺ | ☺ | ☺ | ☹ | ☺ | ☺ | 😐 | ☺ | ☺ | ☺ |
| **Sheehan** | 2009 | ☺ | ☺ | ☺ | ☹ | ☺ | ☹ | 😐 | ☺ | ☺ | ☺ |
| **Thase** | 1997 | 😐 | ☺ | ☺ | ☹ | ☹ | ☺ | 😐 | ☺ | ☺ | ☹ |
| **Tylee** | 1997 | ☺ | ☺ | ☺ | ☹ | ☺ | 😐 | 😐 | ☺ | ☺ | ☹ |
| **Tzanakaki** | 2000 | ☹ | ☺ | ☺ | ☹ | ☺ | ☺ | ☺ | ☺ | ☺ | ☹ |
| **WXL101497** | 2005 | 😐 | ☺ | ☺ | ☹ | ☺ | 😐 | 😐 | ☺ | ☺ | ☺ |
| **De Nayer** | 2002 | 😐 | ☺ | ☺ | ☹ | ☺ | ☺ | 😐 | ☺ | ☺ | ☹ |
| **Fava** | 1998 | 😐 | ☺ | ☺ | ☹ | ☺ | 😐 | 😐 | ☺ | ☺ | ☹ |
| **Keller** | 2007 | ☺ | ☺ | ☺ | ☹ | ☺ | ☺ | 😐 | ☺ | ☺ | ☺ |
| **Silverstone** | 1999 | 😐 | ☺ | ☺ | ☹ | ☺ | ☺ | 😐 | ☺ | ☺ | ☹ |

**Table S1: Studies included and their quality assessment according to the standardized critical appraisal instrument from the Joanna Briggs Institute.**

☹ No

😐 Unclear

☺ Yes

|  | | **Study** | **Response** | | **Remission** | |
| --- | --- | --- | --- | --- | --- | --- |
| **Placebo vs fluoxetine†** | | | **OR** | **[95 % CI]** | **OR** | **[95 % CI]** |
|  | Rudolph* | | 0.73 | [0.42-1.28] | 0.78 | [0.39-1.56] |
|  | Nemeroff* | | 0.75 | [0.43-1.31] | 0.75 | [0.39-1.42] |
|  | Sheehan* | | 1.07 | [0.59-1.92] | 0.98 | [0.46-2.07] |
|  | Silverstone* | | 0.45 | [0.27-0.75] | 0.37 | [0.21-0.65] |
|  | Heiligenstein | | 0.39 | [0.13-1.19] | 0.33 | [0.11-1.03] |
|  | Moreno | | 0.60 | [0.19-1.94] | 0.65 | [0.20-2.14] |
|  | Andreoli | | 0.39 | [0.23-0.64] | 0.44 | [0.26-0.75] |
|  | Bjerkenstedt | | 1.05 | [0.49-2.25] | 0.21 | [0.06-0.67] |
|  | Corrigan | | 0.37 | [0.13-1.00] | . | . |
|  | Fava | | 0.82 | [0.29-2.36] | . | . |
|  | Fava | | . | . | 0.62 | [0.24-1.64] |
|  | Golstein | | 0.84 | [0.37-1.92] | 1.05 | [0.43-2.59] |
|  | MY-1043/BRL-029060/115 | | 0.76 | [0.49-1.16] | . | . |
|  | **Summary measure (MH)** | | **0.65** | **[0.54-0.78]** | **0.56** | **[0.44-0.71]** |
|  | **Summary measure (DSL)** | | **0.65** | **[0.53-0.81]** | **0.57** | **[0.43-0.76]** |
| **Placebo versus venlafaxine†** | | |  |  |  |  |
|  | Rudolph* | | 0.61 | [0.35-1.07] | 0.42 | [0.22-0.81] |
|  | Nemeroff* | | 0.57 | [0.33-1.00] | 0.63 | [0.33-1.19] |
|  | Sheehan* | | 0.60 | [0.33-1.06] | 0.60 | [0.29-1.22] |
|  | Silverstone* | | 0.39 | [0.23-0.65] | 0.37 | [0.21-0.64] |
|  | Cunningham | | 0.30 | [0.18-0.50] | . | . |
|  | Cunningham | | 0.48 | [0.24-0.94] | . | . |
|  | Khan | | 0.51 | [0.32-0.82] | . | . |
|  | Lecrubier | | 0.34 | [0.16-0.73] | . | . |
|  | Mendels | | 0.58 | [0.35-0.98] | . | . |
|  | Rudolph | | 0.43 | [0.26-0.71] | . | . |
|  | Thase | | 0.31 | [0.17-0.57] | 0.45 | [0.23-0.87] |
|  | Guelfi | | . | . | 0.41 | [0.14-1.22] |
|  | WXL101497 | | 0.48 | [0.32-0.73] | 0.47 | [0.31-0.71] |
|  | AK130940 | | 0.52 | [0.35-0.78] | 0.49 | [0.33-0.74] |
|  | **Summary measure (MH)** | | **0.47** | **[0.40-0.54]** | **0.47** | **[0.39-0.58]** |
|  | **Summary measure (DSL)** | | **0.47** | **[0.40-0.54]** | **0.47** | **[0.39-0.58]** |
| **Fluoxetine versus venlafaxine†** | | |  |  |  |  |
|  | Rudolph* | | 0.84 | [0.48-1.45] | 0.53 | [0.29-0.99] |
|  | Nemeroff* | | 0.76 | [0.44-1.32] | 0.84 | [0.46-1.54] |
|  | Sheehan* | | 0.56 | [0.31-0.99] | 0.61 | [0.31-1.23] |
|  | Silverstone* | | 0.86 | [0.51-1.46] | 0.98 | [0.59-1.63] |
|  | Alves | | 0.42 | [0.13-1.31] | 0.28 | [0.09-0.88] |
|  | Clerc | | 0.48 | [0.18-1.28] | . | . |
|  | Costa e Silva | | 0.68 | [0.39-1.19] | 1.00 | [0.66-1.51] |
|  | De Nayer | | 0.45 | [0.23-0.88] | 0.54 | [0.28-1.05] |
|  | Dierick | | 0.62 | [0.39-0.99] | . | . |
|  | Keller | | 1.02 | [0.73-1.43] | 1.03 | [0.78-1.36] |
|  | Tzanakaki | | 0.82 | [0.36-1.85] | 0.81 | [0.37-1.77] |
|  | Tylee | | 1.37 | [0.87-2.16] | 0.93 | [0.60-1.46] |
|  | **Summary measure (MH)** | | **0.8** | **[0.68-0.93]** | **0.86** | **[0.74-1.01]** |
|  | **Summary measure (DSL)** | | **0.77** | **[0.63-0.94]** | **0.83** | **[0.69-1.00]** |

**Table S2: Head to head meta-analyses [using fixed-effects model (Mantel-Haenszel = MH) and random-effects (DerSimonian and Laird = DSL)] of response and remission between 1/ placebo vs fluoxetine 2/ placebo vs venlafaxine and 3/fluoxetine vs venlafaxine**

OR: Odd Ratio

*: Studies comparing venlafaxine and fluoxetine to venlafaxine/fluoxetine placebo

†: No heterogeneity was found

|  | **Fixed effect** | **Random effect** |
| --- | --- | --- |
| **Network meta-analysis of response** |  |  |
| AIC | 9516 | 9537 |
| **Network meta-analysis of remission** |  |  |
| AIC | 6479 | 6498 |

**Table S3: Akaïke’s Information Criterion (AIC) for Fixed effect and random effects meta-analyses of response and remission**

| **FLUp** | | 0.91 | [0.65-1.27] | 1.02 | [0.74-1.41] | **0.54** | **[0.40-0.72]** | | **0.41** | **[0.31-0.55]** |
| --- | --- | --- | --- | --- | --- | --- | --- | --- | --- | --- |
| 0.90 | [0.57-1.41] | **VENLAFp** | | 1.13 | [0.83-1.52] | **0.59** | **[0.47-0.74]** | | **0.45** | **[0.38-0.53]** |
| 1.00 | [0.64-1.55] | 1.11 | [0.76-1.62] | **FLU/VENLAFp** | | **0.53** | **[0.40-0.68]** | | **0.39** | **[0.50-0.75]** |
| **0.51** | **[0.37-0.71]** | **0.56** | **[0.41-0.76]** | **0.50** | **[0.36-0.70]** | **FLU** | | | **0.76** | **[0.64-0.89]** |
| **0.41** | **[0.29-0.59]** | **0.46** | **[0.35-0.59]** | **0.41** | **[0.31-0.54]** | **0.81** | | **[0.68-0.96]** | **VENLAF** | |

**Table S4: Odds ratio (OR) of response and remission between fluoxetine placebo, venlafaxine placebo, fluoxetine/venlafaxine placebo, fluoxetine and venlafaxine.**

**Results of the network meta-analysis (random effect model) are the OR between treatment in the column and treatment in the row with their 95 % confidence interval. For response (in the grey boxes), OR higher than 1 favour the treatment indicated in the row. For remission (in the white boxes), OR higher than 1 favour the treatment indicated in the column. To obtain OR for comparisons in the opposite direction, reciprocals should be taken. Significant results are in bold and underscored.**

**FLUp: fluoxetine placebo**

**FLU/VENLAFp: fluoxetine and venlafaxine placebo**

**VENLAFp: venlafaxine placebo**

**FLU: fluoxetine**

**VENLAF: venlafaxine**

RESPONSE

Fluoxetine versus placebo

REMISSION

Venlafaxine versus placebo

Venlafaxine versus fluoxetine

Fluoxetine versus placebo

Venlafaxine versus placebo

Venlafaxine versus fluoxetine

**Figure S1: Forest plot presenting head-to-head meta-analyses using random-effects model for response and remission comparing 1/ placebo vs fluoxetine 2/ placebo vs venlafaxine and 3/fluoxetine vs placebo**
